# Unusual Change in Murmurs in a Case of Mitral Valve Prolapse

**DOI:** 10.7759/cureus.28411

**Published:** 2022-08-25

**Authors:** Sakiko Honda, Michiyo Yamano, Tatsuya Kawasaki

**Affiliations:** 1 Department of Cardiology, Matsushita Memorial Hospital, Moriguchi, JPN

**Keywords:** preload, phonocardiography, murmur, mitral valve prolapse, echocardiography

## Abstract

Mitral valve prolapse (MVP) has changeable auscultatory features regarding the onset and amplitude with physiologic and pharmacologic maneuvers. We report a case of MVP in which not only the onset and amplitude of systolic murmurs but also the endpoint of systolic murmurs were dynamically altered according to preload. An asymptomatic 61-year-old man presented with a grade 3 crescendo murmur best heard at the apex, which started in the mid-systole without a click, and lasted up to the second sound. A diagnosis of moderate to severe mitral regurgitation due to MVP of P2 scallop was confirmed by echocardiography. At his regular follow-up visit, changes in cardiac auscultation were recognized although the patient was still asymptomatic. A grade 2, high-pitched crescendo murmur, which was softer than the previous findings, started immediately after the first sound and ended approximately 100 ms before the second sound on phonocardiography. On echocardiography, the severity of mitral regurgitation was abated in comparison with the previous findings, and mitral regurgitation abruptly ended in mid-systole but continued to the end of systole during increased preload due to an elevation of the legs. The present case highlights the importance of careful auscultation to estimate hemodynamic conditions in patients with MVP.

## Introduction

Mitral valve prolapse (MVP) has unique auscultatory features characterized by mid-systolic clicks, frequently followed by late systolic murmurs [1,2]. It is well known that clicks and murmurs during systole can change, such as earlier or later onset as well as a louder or softer amplitude, with physiologic and pharmacologic maneuvers [2,3]. We report a case of MVP in which not only the onset and amplitude of systolic murmurs but also the endpoint of systolic murmurs were dynamically altered as a result of decreased preload.

## Case presentation

**Figure 1:**
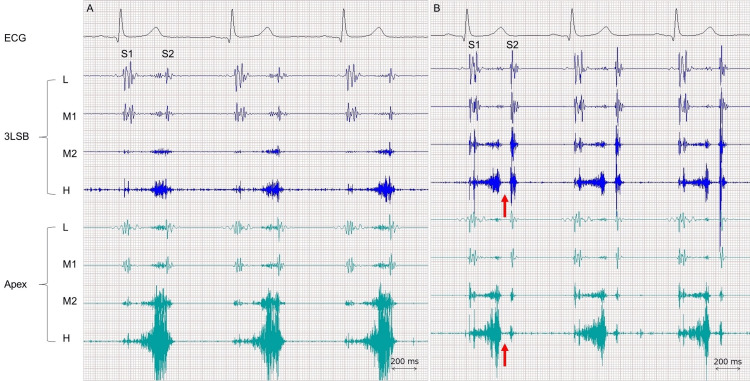
Phonocardiography showing a high-pitched murmur during mid- to late systole, which is louder on the apex than on the third left sternal border (3LSB) on the initial presentation (A). The systolic murmur starts without a click and lasts up to the second sound (S2). Three years later, the high-pitched murmur during systole decreases in intensity (B). Of note, the murmur starts immediately after the first sound (S1) and ends approximately 100 ms before S2 (arrows). Both the phonocardiograms were recorded in the same way in the supine position on the same scale. H = high frequency; L = low frequency: M1 = lower-middle frequency: M2 = higher-middle frequency.

An asymptomatic 61-year-old man was referred to our hospital for heart murmurs after a medical check-up. He had no notable medical history and did not take medication. He stopped smoking at the age of 48 years, drank occasionally, and had no known allergies. The patient reported that there was no family history of cardiovascular diseases. His vital signs and physical examination were normal except for a grade 3 crescendo murmur best heard at the apex, which started in the mid-systole without a click and lasted up to the second sound (Figure 1A). The laboratory data including brain natriuretic peptide were normal and so were an electrocardiogram and chest radiograph.

**Figure 2:**
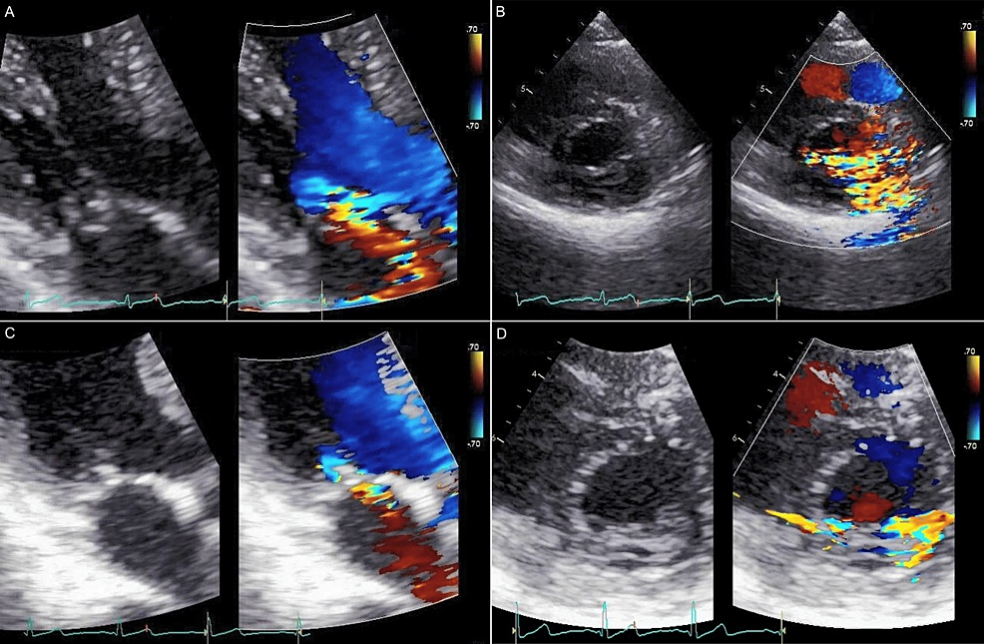
Echocardiography showing mitral regurgitation due to mitral valve prolapse at a presentation on apical three-chamber images (A) and short-axis images (B). The severity of mitral regurgitation improved three years after presentation (C, D).

Echocardiography showed moderate to severe mitral regurgitation due to MVP of the P2 scallop (Figures 2A, 2B). No ruptured chordae tendineae were identified. The left ventricular end-diastolic dimension was 42 mm, the left ventricular ejection fraction was 64%, the left atrial dimension was 31 mm, the peak transmitral E-wave velocity and deceleration time of the transmitral E-wave were 1.19 m/s and 216 ms, respectively, and the transmitral E/A ratio and E/early diastolic mitral annular velocity ratio were 1.61 and 13.37, respectively. Surgical repair was deferred since neither pulmonary hypertension nor myocardial ischemia was provoked immediately after exercise on a treadmill at a maximal workload, heart rate, and double product of 10.2 metabolic equivalents, 161 bpm, and 28,336 bpm-mmHg, respectively. The left atrial volume index before exercise was 45 mL/m^2^ (reference, 17 to 32).

**Figure 3:**
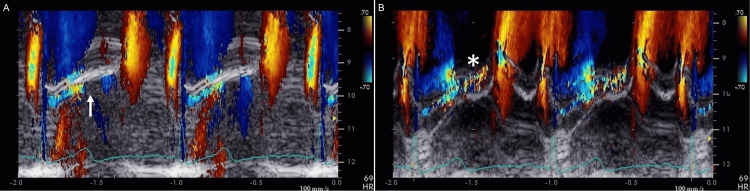
Echocardiography before and after leg elevation Color-Doppler M-mode images obtained in the left lateral decubitus position show that the mosaic signal of mitral regurgitation ends in the mid-systole before an elevation of the legs (A, arrow). After the leg lift, the mosaic signal extends to the end of the systole or continues until the start of the left ventricular inflow (B, asterisk).

The patient was followed for an interval of six months, and no change was noted regarding mitral regurgitation due to MVP. Three years after presentation, however, changes in cardiac auscultation were recognized although the patient was still asymptomatic. A grade 2, high-pitched crescendo murmur, which was softer than the previous findings, started immediately after the first sound and ended in the mid-systole (Figure 1B). Echocardiography revealed moderate mitral regurgitation due to MVP of P2 scallop (Figures 2C, 2D); the severity of mitral regurgitation was abated in comparison with the previous findings. The left ventricular end-diastolic dimension was 41 mm, the left ventricular ejection fraction was 64%, and the left atrial dimension was 29 mm; findings almost unchanged from the previous data, but the left atrial volume index was smaller than the previous value (i.e., 22 mL/m^2^). Furthermore, the heart rates during echocardiography were faster than the first examination by 8-10 bpm, although no treatment including diuretics was initiated. Of note, mitral regurgitation abruptly ended in mid-systole in the supine position but continued to the end of systole during increased preload due to an elevation of the legs (Figures 3A, 3B).

Based on the asymptomatic status and his preference, neither drug initiation nor surgery was done. The patient was doing well for more than four years after the presentation.

## Discussion

The present patient was referred for heart murmurs and was diagnosed with MVP accompanied by mitral regurgitation. On auscultation, the features of the murmurs were typical of MVP, such as late systolic, medium to high pitched, and best heard at the apex [1,2] although clicks were lacking. Changes in auscultatory findings were noticed on the follow-up visit; of note, not only the onset and amplitude of the murmurs but also the endpoint moved toward the first sound, which was confirmed by phonocardiography and was associated with decreased preload as assessed by echocardiography.

Systolic clicks are thought to develop at the onset of MVP and can be modified with physiologic and pharmacologic maneuvers [2,3]. For example, clicks are accelerated with inspiration and standing, whereas beta-blockers and handgrip result in delayed onset [4,5]. This phenomenon is likely to be explained by changes in the left ventricular volume and contractility induced by these maneuvers [4]. In addition, late systolic murmurs due to mitral regurgitation after clicks can occur earlier or later and become louder or softer according to the maneuver [5]. Although the systolic clicks were still unclear in the current case, the systolic murmurs started approximately 100 ms earlier and became softer than the previous measurements, findings that have not been reported to our knowledge. The auscultatory changes may be explained by the smaller left ventricular end-diastolic dimension as assessed by echocardiography. It is reported that murmurs in patients with MVP become softer and occur earlier immediately after inhalation of amyl nitrate decreases the heart size [2].

Patients with MVP can have late-systolic murmurs or pan-systolic murmurs with late systolic accentuation; the latter seems more severe regurgitation than the former [2,6]. It is worth noting that these systolic murmurs persist to or slightly beyond the aortic component of the second sound (i.e., the isovolumic relaxation time) [6]; findings consistent with the current case on the initial examination. However, on the follow-up visit three years after the presentation, systolic murmurs on auscultation ended before the second sound, which was clearly perceived. Phonocardiography demonstrated that the systolic murmurs abruptly ended approximately 100 ms before the second sound. This phenomenon is extremely unusual since murmurs are likely to continue up to the second sound regardless of the effects of the maneuvers on systolic murmurs in patients with MVP [5].

An early end of mitral regurgitation signals was confirmed by echocardiography, which was performed immediately after the phonocardiography on the follow-up visit. It is reasonable to consider decreased preload as a possible underlying mechanism, given that the early end of the systolic murmur disappeared with the maneuver of increased preload (i.e., leg lift). Decreased left ventricular end-diastolic volume as well as decreased left atrial volume index may also support the speculation. However, attention should be paid to the relation between regurgitation signals and murmurs since echocardiography can detect more sensitively minute changes in frequency range than vibrations in the audible frequency range [7]. Auscultatory findings on systolic murmurs seem to underestimate mitral regurgitation due to MVP regarding its timing. In a detailed analysis using phonocardiography in patients with MVP, not late systolic murmurs but early systolic murmurs can be infrequently recorded although the duration of mitral regurgitation was pansystolic and up to the isovolumic relaxation time on echocardiography [6]. Further studies are required to evaluate the frequency and clinical significance of the endpoint of systolic murmurs in patients with MVP.

## Conclusions

MVP is known to show changes in auscultatory features, e.g., earlier or later onset of the murmurs, with physiologic and pharmacologic maneuvers. The present case highlights the importance of careful auscultation, not only with regard to the onset timing but also with regard to the end timing of systolic murmurs, to estimate the hemodynamic conditions in patients with MVP.
